# Efficacy and safety analysis of neoadjuvant chemotherapy combined with immunotherapy in patients with muscle-invasive bladder cancer

**DOI:** 10.3389/fimmu.2024.1479743

**Published:** 2024-11-01

**Authors:** Yanhang Yu, Chuanao Zhang, Hao Chen, Jianglei Zhang, Jun Ouyang, Zhiyu Zhang

**Affiliations:** Department of Urology, The First Affiliated Hospital of Soochow University, Suzhou, China

**Keywords:** muscle-invasive bladder cancer, neoadjuvant chemotherapy, immunotherapy, efficacy, safety

## Abstract

**Introduction:**

This study examined the efficacy and safety of neoadjuvant chemotherapy combined with immunotherapy in patients with muscle-invasive bladder cancer (MIBC).

**Methods:**

This retrospective cohort study included patients diagnosed with MIBC at the First Affiliated Hospital of Soochow University between January 1, 2020, and December 31, 2023, assigned to either chemotherapy (gemcitabine with cisplatin) or combination (chemotherapy plus toripalimab or tislelizumab) groups based on the neoadjuvant treatment regimen. Key metrics, including pathological downstaging rate (PDR), pathological complete response rate (PCRR), and incidence and severity of adverse events (AEs), were compared between groups.

**Results:**

This study included 53 patients (mean age: 67.21 years). In the combination group, 14 patients (51.85%) achieved pathological complete remission (ypT0), and seven (25.93%) achieved partial remission (ypT1), resulting in a PDR and PCRR of 77.78 and 51.85%, respectively. In the chemotherapy group, six patients (23.08%) achieved complete remission, and five (19.23%) achieved partial remission, resulting in a PDR and PCRR of 42.31 and 23.08%, respectively. Differences between groups were statistically significant (p < 0.05). There were no significant differences in pathological downstaging or complete remission rates among subgroups in the combination group (p > 0.05). No serious allergic reactions or fatal AEs were detected in either group, with no grade 4 AEs. Grade 3 AE rates were 22.22 and 20.83% in the combination and chemotherapy groups, respectively, although non-significant (p > 0.05).

**Conclusion:**

Neoadjuvant chemotherapy combined with immunotherapy had enhanced efficacy and manageable safety in patients with MIBC, suggesting its potential for integration into clinical practice.

## 1 Introduction

Bladder cancer is a prevalent malignancy with high morbidity and mortality rates, affecting 430,000 individuals worldwide annually (1). It ranks as the fourth most common cancer among males and the tenth among females (2). Muscle-invasive bladder cancer (MIBC) is particularly challenging owing to its aggressive nature and tendency to metastasize (3). Approximately 30% of urothelial bladder cancers are classified as MIBC (4). Currently, the standard treatment for MIBC (T2~T4aN0M0) includes at least three cycles of platinum-based neoadjuvant chemotherapy (NAC) followed by radical cystectomy (RC) (5, 6). Despite this aggressive approach (NAC + RC), over 40% of patients with MIBC experience recurrence or death within three years (5, 6). Moreover, NAC only improves the 5-year survival rate by 5 to 8%, with approximately 40% of patients responding to NAC (4). Therefore, there is an urgent need to develop new neoadjuvant treatment strategies to improve outcomes in patients with MIBC.

Recently, immune checkpoint inhibitors (ICIs), including anti-programmed cell death protein-1 (PD-1)/programmed death ligand-1 (PD-L1) agents, have shown promising survival benefits in patients with locally advanced and metastatic bladder cancer (7, 8). However, whether combining NAC with immunotherapy would result in enhanced efficacy in patients with MIBC warrants further investigation. In the current study, we included 53 patients with MIBC who were treated at the First Affiliated Hospital of Soochow University. We analyzed the pathological complete response rate (PCRR), pathological downstaging rate (PDR), and incidence of adverse events (AEs) for different neoadjuvant treatment regimens to evaluate the efficacy and safety of combined chemotherapy and immunotherapy.

## 2 Materials and methods

### 2.1 Study design

This was a retrospective cohort study.

### 2.2 Patient selection

This study included patients with MIBC treated at the First Affiliated Hospital of Soochow University between January 2020 and December 2023. Inclusion criteria were as follows: (1) MIBC diagnosis confirmed by transurethral resection of bladder tumor (TURBT) pathology, (2) receiving at least three cycles of neoadjuvant therapy, and (3) tumor stage T2–T4aN0M0. Exclusion criteria were as follows: (1) presence of other malignancies or infectious diseases, (2) not undergoing RC after neoadjuvant therapy, (3) metastatic bladder cancer, and (4) incomplete clinical data or refusal to participate in the study. The study was conducted in accordance with the Declaration of Helsinki (2013 revision) and approved by the Ethics Committee of the First Affiliated Hospital of Soochow University (Approval ID: No.484, 2024). All patients signed informed consent forms before participation.

### 2.3 Treatment regimen

In our study, all patients underwent immediate postoperative intravesical instillation therapy following TURBT. Specifically, each was administered 50 mg of epirubicin as part of the post-surgical treatment protocol. Once pathology confirmed MIBC, patients were grouped according to the treatment regimen into two categories: 26 patients who received gemcitabine and cisplatin (GC) NAC (chemotherapy group), and 27 patients who received GC combined with PD-1 inhibitor immunotherapy (neoadjuvant immune checkpoint blockade, NICB) (combination group). In the combination treatment group, 16 and 11 patients received toripalimab and tislelizumab, respectively.

The GC regimen comprised gemcitabine 1000 mg/m², administered intravenously on days 1 and 8, and cisplatin70 mg/m², administered intravenously on days 2–3 to reduce chemotherapy reactions and improve tolerance. The immunotherapy regimen comprised either toripalimab 200 mg or tislelizumab 240 mg, administered intravenously on day 8. Each treatment cycle lasted for 21 days, with patients undergoing at least three cycles prior to surgery. Before each treatment, routine blood tests, biochemical tests, thyroid function tests, and levels of cardiac markers and adrenal hormones were monitored. Multiparametric magnetic resonance imaging or enhanced computed tomography was performed prior to surgery, and clinical data were collected during outpatient visits, from hospital records, or by conducting telephonic follow-ups.

### 2.4 Observation metrics

The primary endpoint of this study was the postoperative PDR, whereas the secondary endpoints were the incidence of AEs during treatment and PCRR. Pathological downstaging was defined as the absence of bladder muscle invasion and lymph node metastasis (≤ ypT1N0M0) in postoperative pathology, and pCR was defined as the absence of tumor residual (ypT0N0M0) in postoperative pathology. AEs were recorded and evaluated according to the Common Terminology Criteria for Adverse Events (CTCAE) version 5.0 during neoadjuvant therapy (9).

### 2.5 Statistical analysis

Data analyses were conducted using R version 4.2.1, specifically utilizing the ‘stats[4.2.1]’ package. Categorical data are presented as frequencies (%), whereas continuous data are expressed as mean ± standard deviation. For numerical variables, if the data met the assumptions of a normal distribution and homogeneity of variance, a t-test was used for group comparisons. If the data were normally distributed but did not meet the homogeneity of variance assumption, Welch’s t-test was used. Non-normally distributed data were compared using the Wilcoxon test. For categorical variables, if the expected frequency was > 5 and the total sample size was ≥ 40, a chi-square test was used for group comparisons. If the expected frequency was between 1 and 5 with a total sample size of ≥ 40, Yates’ correction for continuity was applied. Fisher’s exact test was used if the expected frequency was < 1 or the total sample size was < 40, Fisher’s exact test was used. A p-value of < 0.05 was considered statistically significant.

## 3 Results

### 3.1 Demographic statistics

Both groups showed no statistically significant differences between collected baseline data, including age, sex, body mass index (BMI), hypertension, diabetes, hyperlipidemia, smoking history, Eastern Cooperative Oncology Group (ECOG) score, histological grade, and preoperative clinical stage (p > 0.05) (**Table 1**). According to preoperative clinical staging, 59.26% (16/27) of patients in the combination group and 69.23% (18/26) of patients in the chemotherapy group had cT3-stage tumors.

**Table 1:** Comparison of demographic characteristics between the combination and chemotherapy groups.

Characteristics	Combination Group	Chemotherapy Group	p-value
n	27	26	
Sex, n (%)			0.776
Male	21 (77.78%)	22 (84.62%)	
Female	6 (22.22%)	4 (15.38%)	
Age (years), mean ± SD	67.59 ± 7.82	66.81 ± 11.91	0.777
BMI (kg/m^2^), mean ± SD	23.42 ± 2.94	23.46 ± 3.52	0.960
Hypertension, n (%)			0.020
Yes	8 (29.63%)	16 (61.54%)	
No	19 (70.37%)	10 (38.46%)	
Diabetes, n (%)			0.078
Yes	3 (11.11%)	8 (30.77%)	
No	24 (88.89%)	18 (69.23%)	
Hyperlipidemia, n (%)			0.691
Yes	6 (22.22%)	7 (26.92%)	
No	21 (77.78%)	19 (73.08%)	
Smoking history, n (%)			0.697
Yes	8 (29.63%)	9 (34.62%)	
No	19 (70.37%)	17 (65.38%)	
ECOG score, n (%)			0.379
0	11 (40.74%)	13 (50.00%)	
1	12 (44.44%)	7 (26.92%)	
2	2 (7.41%)	5 (19.23%)	
3	2 (7.41%)	1 (3.85%)	
Histological grade, n (%)			0.370
High Grade	23 (85.19%)	25 (96.15%)	
Low Grade	4 (14.81%)	1 (3.85%)	
cT staging, n (%)			0.713
cT2	11 (40.74%)	8 (30.77%)	
cT3	8 (29.63%)	8 (30.77%)	
cT4a	8 (29.63%)	10 (38.46%)	

BMI, body mass index; ECOG, Eastern Cooperative Oncology Group.

### 3.2 Comparison of PDR and PCRR between the two groups

In the combination group, 14 patients achieved complete remission (ypT0N0M0), while seven achieved partial remission (ypT1/Ta/TisN0M0), resulting in a PDR of 77.78% and a PCRR of 51.85%. In contrast, the chemotherapy group had six patients who achieved complete remission (ypT0N0M0) and five who achieved partial remission (ypT1/Ta/TisN0M0), resulting in a PDR of 42.31% and a PCRR of 23.08%. The PDR and PCRR of the combination group were significantly higher than those of the chemotherapy group, with statistically significant differences (p < 0.05), as shown in **Table 2**.

**Table 2:** Comparison of pathological downstaging rate and pathological complete response rate between the combination and chemotherapy groups.

Characteristics	Combination Group	Chemotherapy Group	p-value
n	27	26	
Pathological outcome, n (%)			0.025
ypT0	14 (51.85%)	6 (23.08%)	
ypT1/Tis/Ta	7 (25.93%)	5 (19.23%)	
≥ypT2	6 (22.22%)	15 (57.69%)	
PCRR, n (%)			0.031
Yes	14 (51.85%)	6 (23.08%)	
No	13 (48.15%)	20 (76.92%)	
PDR, n (%)			0.008
Yes	21 (77.78%)	11 (42.31%)	
No	6 (22.22%)	15 (57.69%)	

PDR, pathological downstaging rate; PCRR, pathological complete response rate.

### 3.3 Comparison of PDR and PCRR within subgroups of the combination group

In the combination group, 16 patients received toripalimab 200 mg, and 11 patients received tislelizumab 240 mg. Within the toripalimab subgroup, 10 patients achieved complete remission (ypT0N0M0), and 2 achieved partial remission (ypT1/Ta/TisN0M0), resulting in a PDR of 75.00% and a PCRR of 62.50%. In the tislelizumab subgroup, four patients achieved complete remission (ypT0N0M0), while five achieved partial remission (ypT1/Ta/TisN0M0), resulting in a PDR of 81.82% and a PCRR of 36.36%. There were no statistically significant differences between the two subgroups in terms of PDR or PCRR (p > 0.05) (**Table 3**).

**Table 3:** Comparison of pathological downstaging rate and pathological complete response rate within subgroups of the combination group.

Characteristics	Toripalimab	Tislelizumab	p-value
n	16	11	
Pathological outcome, n (%)			0.199
ypT0	10 (62.50%)	4 (36.36%)	
ypT1/Tis/Ta	2 (12.50%)	5 (45.45%)	
≥ypT2	4 (25.00%)	2 (18.18%)	
PCRR, n (%)			0.252
Yes	10 (62.50%)	4 (36.36%)	
No	6 (37.50%)	7 (63.64%)	
PDR, n (%)			1.000
Yes	12 (75.00%)	9 (81.82%)	
No	4 (25.00%)	2 (18.18%)	

PDR, pathological downstaging rate; PCRR, pathological complete response rate.

### 3.4 Incidence of short- to medium-term AEs

Neither group experienced severe allergic reactions or fatal AEs, and no grade 4 AEs were reported. The combination group had a generally higher incidence of AEs, such as rash, gastrointestinal reactions, hematologic toxicity, endocrine dysfunction, and liver and kidney function damage, than the chemotherapy group. The incidence of grade 3 AEs was 29.63% (8/27) and 19.23% (5/26) in the combination and chemotherapy groups, respectively. However, the difference in the incidence of grade 3 AEs between the two groups was not statistically significant (p > 0.05; **Table 4**).

**Table 4:** Comparison of adverse event rates between the combination and chemotherapy groups.

Characteristics	Combination Group	Chemotherapy Group	p-value
n	27	26	
Rash, n (%)			0.393
0	22 (81.48%)	24 (92.31%)	
1	4 (14.81%)	1 (3.85%)	
2	1 (3.70%)	1 (3.85%)	
Gastrointestinal reactions, n (%)			0.850
0	14 (51.85%)	15 (57.69%)	
1	9 (33.33%)	7 (26.92%)	
2	2 (7.41%)	3 (11.54%)	
3	2 (7.41%)	1 (3.85%)	
Liver and kidney function damage, n (%)			0.361
0	22 (81.48%)	23 (88.46%)	
1	2 (7.41%)	0 (0.00%)	
2	0 (0.00%)	1 (3.85%)	
3	3 (11.11%)	2 (7.69%)	
Hematologic toxicity, n (%)			0.333
0	17 (62.96%)	19 (73.08%)	
1	4 (14.81%)	5 (19.23%)	
2	3 (11.11%)	0 (0.00%)	
3	3 (11.11%)	2 (7.69%)	
Endocrine dysfunction, n (%)			0.513
0	24 (88.89%)	25 (96.15%)	
1	2 (7.41%)	1 (3.85%)	
2	1 (3.70%)	0 (0.00%)	
Total adverse events, n (%)			0.898
Grade 1–2	28	19	
Grade 3	8	5	

## 4 Discussion

In patients with bladder cancer, the potential survival benefit of chemotherapy combined with immunotherapy remains controversial. Phase III trials have yielded conflicting results (8, 10). The IMvigor130 study demonstrated that atezolizumab combined with platinum-based chemotherapy substantially improved progression-free survival in patients with metastatic MIBC (8). However, the KEYNOTE-361 trial revealed that pembrolizumab combined with platinum-based chemotherapy did not markedly improve progression-free survival or overall survival in patients with advanced bladder cancer (10). These trials included patients who did and did not meet the criteria for cisplatin eligibility, and the majority of participants in both studies received carboplatin-based chemotherapy. Therefore, these results cannot be extrapolated to patients with MIBC who are eligible for cisplatin therapy.

The CheckMate-901 study indicated that nivolumab combined with GC chemotherapy provided a survival benefit in patients with advanced bladder cancer who were cisplatin-tolerant (11). Compared to traditional NAC, the addition of NICB may potentially re-activate or enhance anti-tumor immunity in peripheral blood and the tumor microenvironment. This can be achieved by activating, proliferating, and releasing tumor-specific cytotoxic T-cells into the bloodstream, as well as by enhancing the presentation of tumor antigens by dendritic cells to naive T-cells in tumor-draining lymph nodes. These processes collectively promote systemic anti-tumor immunity, thereby targeting micro-metastases or circulating tumor cells that could cause postoperative recurrence (12). In both cancer cell lines and mouse models, cisplatin has been shown to induce upregulation of PD-L1 expression, increase T-cell infiltration, and transform tumors into “hot” tumors (13). Ramakrishnan et al. (14) found that cisplatin could enhance the cytotoxic effects of T-cells by upregulating mannose-6-phosphate receptor expression on tumor cells, making it easier for granzyme B released by activated cytotoxic T-cells to penetrate tumor cells. These findings demonstrate that cisplatin can enhance the efficacy of immunotherapy. In contrast, carboplatin, owing to its strong myelosuppressive effects, causes considerable lymphocyte destruction and may inhibit cytotoxic T-lymphocytes in the tumor microenvironment (15). Therefore, cisplatin may be a better choice when selecting platinum-based chemotherapy drugs for combination with immunotherapy.

Currently, only a few prospective clinical trials have demonstrated the efficacy and safety of standalone neoadjuvant immunotherapy or NICB (16, 17). Funt et al. (18) applied atezolizumab combined with GC as a neoadjuvant therapy in patients with MIBC; however, their cohort primarily consisted of cT2-stage patients (79.49%), lacking efficacy results for advanced bladder cancer in the neoadjuvant setting. Recently, the findings of the Phase 3 NIAGARA trial underscore the effectiveness of incorporating durvalumab with neoadjuvant chemotherapy in MIBC (19). The results reveal enhanced event-free survival (EFS) and overall survival (OS) rates compared to chemotherapy alone, with EFS HR 0.68 and OS HR 0.75. Similar to the previous study, cT2-stage patients made up 40.3% of the participants, again indicating a lack of efficacy results for advanced bladder cancer in the neoadjuvant setting. In our study, 64.15% of patients were clinically stage cT3 or higher, and the PDR and PCRR were comparable, confirming its safe application. Compared with previous prospective clinical trials for patients with MIBC, which reported a PCRR of 30–36% and a PDR of 50–66%, our study demonstrated slightly better outcomes for both PCRR and PDR. Although the incidence of AEs increased, no grade 4 AEs were observed. Overall, NICB demonstrated good efficacy and safety. Most existing NICB clinical trials are single-arm studies with PCRRs ranging between 37 and 46%, and direct randomized controlled trials comparing different immunotherapy drugs for neoadjuvant treatment in bladder cancer are still lacking (20). Toripalimab, an engineered PD-1 antibody with an improved Fc segment eliminating antibody-dependent phagocytosis, exhibits strong anti-tumor activity (21). Tislelizumab has shown superior efficacy in clinical trials as a second-line treatment for advanced urothelial carcinoma (22). As the only two approved ICIs for urothelial carcinoma in China, they offer economic efficiency, although further comparative studies are needed to confirm their selection in neoadjuvant therapy.

Based on the results of this study, NICB appears to be more effective than conventional NAC. However, there are currently no effective biomarkers for identifying patients who are most likely to benefit from this treatment. The antitumor efficacy of immunotherapy does not always correlate with PD-L1 expression, and tumors with low PD-L1 expression can respond to immunotherapy. In some clinical studies, pathological staging has been identified as an independent predictor of efficacy, along with other hematological indicators that reflect systemic inflammation, such as hemoglobin, platelets, globulin, and the platelet-to-lymphocyte ratio (23). Clinical trials are exploring more objective, non-invasive biomarkers such as DNA mutations and methylation in the blood and urine to assess the presence of residual tumor cells (24, 25).

The current study demonstrates that NICB can substantially enhance the PDR in patients with MIBC, reaching 77.78%, and shows a higher PCRR than GC-based NAC without notably increasing the incidence of grade 3 to 4 treatment-related AEs, indicating its safe application. However, the number of patients with NICB included in this study was relatively small, and long-term follow-up data are lacking. Future investigations should include a larger patient cohort and long-term follow-up to evaluate the impact on long-term survival benefits. Besides, this study’s retrospective design poses a risk of selection bias due to non-randomized treatment assignments. Future improvements include employing randomized controlled trials and detailing patient selection criteria to minimize bias and enhance result validity. Additionally, further large-sample studies are needed to develop sensitive biomarkers to accurately identify patients who are most likely to benefit from this treatment.

## Data Availability

The datasets presented in this study can be found in online repositories. The names of the repository/repositories and accession number(s) can be found below: https://figshare.com/s/248759cfcbf04a10b171.
